# From Control to Cure: Insights into the Synergy of Glycemic and Antibiotic Management in Modulating the Severity and Outcomes of Diabetic Foot Ulcers

**DOI:** 10.3390/ijms26146909

**Published:** 2025-07-18

**Authors:** Idris Ajibola Omotosho, Noorasyikin Shamsuddin, Hasniza Zaman Huri, Wei Lim Chong, Inayat Ur Rehman

**Affiliations:** Department of Clinical Pharmacy and Pharmacy Practice, Faculty of Pharmacy, Universiti Malaya, Kuala Lumpur 50603, Malaysia; s2003667@siswa.um.edu.my (I.A.O.); noorasyikin@um.edu.my (N.S.); williamchongwl@um.edu.my (W.L.C.); inayat.rehman@um.edu.my (I.U.R.)

**Keywords:** diabetic foot ulcer, advanced glycation end products (AGEs), antihyperglycemic agents, antibiotic therapy, reactive oxygen species (ROS), glycemic control, macrophage polarization (M1/M2 Transition)

## Abstract

Diabetic foot ulcers (DFUs), which affect approximately 15% of individuals with diabetes mellitus (DM), result from complex molecular disturbances involving chronic hyperglycemia, immune dysfunction, and infection. At the molecular level, chronic hyperglycemia promotes the formation of advanced glycation end products (AGEs), activates the AGE-RAGE-NF-κB axis, increases oxidative stress, and impairs macrophage polarization from the pro-inflammatory M1 to the reparative M2 phenotype, collectively disrupting normal wound healing processes. The local wound environment is further worsened by antibiotic-resistant polymicrobial infections, which sustain inflammatory signaling and promote extracellular matrix degradation. The rising threat of antimicrobial resistance complicates infection management even further. Recent studies emphasize that optimal glycemic control using antihyperglycemic agents such as metformin, Glucagon-like Peptide 1 receptor agonists (GLP-1 receptor agonists), and Dipeptidyl Peptidase 4 enzyme inhibitors (DPP-4 inhibitors) improves overall metabolic balance. These agents also influence angiogenesis, inflammation, and tissue regeneration through pathways including AMP-activated protein kinase (AMPK), mechanistic target of rapamycin (mTOR), and vascular endothelial growth factor (VEGF) signaling. Evidence indicates that maintaining glycemic stability through continuous glucose monitoring (CGM) and adherence to antihyperglycemic treatment enhances antibiotic effectiveness by improving immune cell function and reducing bacterial virulence. This review consolidates current molecular evidence on the combined effects of glycemic and antibiotic therapies in DFUs. It advocates for an integrated approach that addresses both metabolic and microbial factors to restore wound homeostasis and minimize the risk of severe outcomes such as amputation.

## 1. Introduction

Diabetic foot ulcers (DFUs) are a serious and debilitating complication of diabetes mellitus, affecting up to 15% of individuals with the condition during their lifetime and accounting for 20–30% of diabetes-related hospitalizations [1,2]. Even minor foot trauma can develop into a DFU, with approximately 15% of these cases leading to amputation [1]. Characterized by a rupture of the epidermis and dermis, DFUs result from a complex interaction of neuropathy, peripheral arterial disease (PAD), and weakened immune function, often worsened by uncontrolled hyperglycemia [3]. However, accumulating evidence shows that these clinical outcomes are driven by molecular disruptions affecting tissue regeneration, immune regulation, and host–microbe balance [4,5,6].

Emerging molecular evidence shows that chronic hyperglycemia triggers a series of pathological pathways. These include the formation of advanced glycation end products (AGEs), which activate the AGE-RAGE-NF-κB pathway, increasing oxidative stress, and the ongoing activation of inflammatory mediators that impair keratinocyte and macrophage function, endothelial stability, and fibroblast activity in wound environments [5]. These processes result in delayed re-epithelialization, disrupted immune cell transitions (especially M1-to-M2 macrophage polarization), and excessive production of reactive oxygen species (ROS), all of which hinder granulation tissue formation and impair angiogenesis [7]. This persistent inflammatory state causes tissue damage and prevents wound healing, highlighting not only the importance of glycemic control but also the need for therapeutic strategies that target redox balance, inflammatory modulation, and cellular repair mechanisms in DFU care [8].

DFU management becomes more complicated due to infections, which often involve both Gram-positive bacteria (e.g., Staphylococcus aureus and Enterococcus faecalis) and Gram-negative bacteria (e.g., Escherichia coli and Klebsiella pneumoniae). These infections worsen tissue damage and delay healing [4,9]. Effective management requires a dual approach: strict glycemic control to support metabolic stability and wound healing, along with culture-guided antibiotic therapy to eliminate infection [10]. Continuous glucose monitoring (CGM) has become a crucial tool for achieving optimal glycemic control, offering real-time glucose level tracking and enabling timely treatment adjustments. However, challenges such as delayed medical intervention—often due to cultural dependence on traditional healing practices—and poor adherence to clinical guidelines contribute to poor outcomes, including gangrene and amputation [11,12,13,14]. Poor glycemic control weakens the immune response by decreasing phagocytic activity, increasing pathogen virulence, and setting off a cycle that worsens tissue damage [4,15,16,17]. Furthermore, conditions like osteomyelitis and soft tissue cellulitis are not just surface issues; they often indicate deeper problems in immune signaling and tissue metabolism [15,16].

While the role of glycemic control in wound healing is well recognized, its molecular interactions remain insufficiently studied [18]. Literature mainly discusses these treatments separately, with limited attention to their potential combined effects in clinical practice. Increasing evidence indicates that maintaining stable glycemic levels can enhance antibiotic effectiveness by aiding immune system recovery, reducing oxidative stress, and disrupting bacterial quorum-sensing mechanisms [15,16,17]. In addition to lowering blood glucose, antihyperglycemic drugs like metformin, GLP-1 receptor agonists, and DPP-4 inhibitors have broader biological effects. These include activating AMPK, inhibiting mTOR, and increasing the expression of VEGF and NGF pathways that support angiogenesis, decrease inflammation, and promote tissue repair—all highly relevant to healing diabetic foot ulcers [19,20]. Furthermore, inadequate patient education, poor treatment adherence, and limited access to comprehensive foot care further complicate the management of DFUs [6].

This review aims to provide a comprehensive molecular view of DFU pathogenesis and management. By integrating current findings on hyperglycemia-related molecular pathways, immune responses, microbial interactions, and the impacts of glycemic and antibiotic treatments, we introduce a translational model that connects molecular research with practical clinical application. It intends to offer valuable insights for clinicians to optimize DFU management, reduce DFU-related complications, prevent amputations, and ultimately lessen the burden on healthcare systems [21,22,23].

## 2. Pathophysiology of Diabetic Foot Ulcers

**Figure 1:**
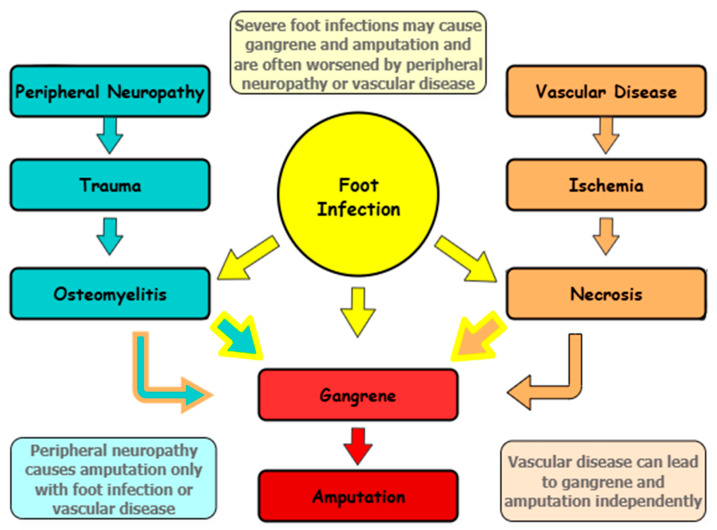
The Triad of Diabetic Foot Complications: neuropathy (causing trauma), PAD (causing ischemia), and infection (driving inflammation) interact to promote DFU progression. Foot infection and vascular disease can develop into gangrene and ultimately require amputation. Peripheral neuropathy leads to amputation only if foot infection (blue/yellow arrow) or vascular disease are present (blue/brown arrow).

DFUs result from a complex interaction of metabolic, neurological, and vascular abnormalities in both type 1 diabetes mellitus (T1DM) and type 2 diabetes mellitus (T2DM) [24]. Peripheral neuropathy, PAD, ischemia, hyperglycemia, and infections lead to tissue breakdown, delayed wound healing, and severe complications such as osteomyelitis (bone infection) [24]. This section examines these mechanisms, highlighting their combined role in DFU progression, as shown in Figure 1, which illustrates how neuropathy, PAD, and infection contribute to DFU severity [25].

### 2.1. Neuropathy, Peripheral Arterial Disease, and Ischemia

Peripheral neuropathy, a complication of chronic diabetes, leads to muscle wasting and foot deformities, which increase focal pressure and the risk of DFU [24,25]. It causes structural foot changes, such as hammer toes, that raise pressure on specific areas of the foot. These changes, especially when combined with poor foot care, raise the likelihood of skin breakdown [26]. PAD further complicates DFU development by reducing blood flow to the lower limbs and promoting ulcer formation [27]. PAD decreases oxygen and nutrient supply, resulting in ischemia and tissue necrosis, with its progression mainly driven by endothelial dysfunction, arterial stiffness, and oxidative stress [27].

If not managed promptly, DFUs follow a predictable progression [24]: neuropathy increases the risk of trauma and bone infections like osteomyelitis. At the same time, reduced blood flow from PAD can lead to ischemia and eventual tissue death. If untreated, ischemia can develop into gangrene, significantly increasing the severity of trauma and ulcer formation [24].

The presence of infection can further accelerate tissue deterioration, highlighting the importance of comprehensive management strategies, including proper glycemic control and appropriate antibiotic therapy to break this cycle and improve clinical outcomes for individuals with DFUs. This is shown in Figure 1.

### 2.2. Hyperglycemia, Infection, and Inflammation

Chronic hyperglycemia impairs wound healing by disrupting immune function and microvascular blood flow, reducing oxygen and nutrient delivery, and increasing the risk of infection [28]. It suppresses nitric oxide production, which heightens oxidative stress and inflammation, and activates Protein Kinase C (PKC), worsening vascular dysfunction and neuropathy [29]. Hyperglycemia also increases metalloproteinase activity, degrading tissue and delaying wound closure, resulting in fragile granulation tissue [30].

The development of DFU centers on immune dysfunction, neuropathy, angiopathy, and metabolic disturbances [29]. Infections sustain the severity of DFU through ongoing inflammatory responses. Pathogens such as Staphylococcus aureus and Escherichia coli invade the epithelium, triggering cytokine release and macrophage activation [30]. In hyperglycemia, bacterial biofilms—protective communities resistant to antibiotics—persist, increasing inflammation via pro-inflammatory M1 macrophages. This cycle raises the risk of osteomyelitis and amputation, with severe infections linked to a 40% five-year amputation rate [30]. Additionally, poor glycemic control encourages colonization by drug-resistant pathogens, complicating treatment and worsening outcomes [30].

### 2.3. From Hyperglycemia to Impaired Healing: Molecular Insights into DFU Pathogenesis

The development of DFUs occurs due to diabetes-related chronic hyperglycemia, which triggers multiple molecular pathways. The Maillard reaction between reducing sugars and proteins, peptides, or amino acids results in the formation of AGEs through non-enzymatic glycation of these molecules [15]. The reaction is accelerated by oxidative stress, ongoing hyperglycemia, and dyslipidemia [31].

The accumulation of AGEs in T2DM patients serves as both a biomarker for glycemic damage and a mechanism explaining the persistence of metabolic memory effects from previous hyperglycemia [31]. The decrease in AGE levels during sustained glycemic control indicates their function as reversible pathogenic mediators [15]. AGEs cause harmful cellular effects by binding to the Receptor for Advanced Glycation End Products (RAGE), which activates mitogen-activated protein kinase (MAPK), extracellular signal-regulated kinase (ERK), Janus kinase (JAK), nuclear factor kappa B (NF-κB), transforming growth factor-alpha (TGF-α), and Nicotinamide Adenine Dinucleotide Phosphate (NADPH) oxidase-1 (NOX-1) signaling pathways [15]. The signaling cascade results in the production of reactive oxygen species (ROS), which damage DNA, proteins, and lipids through oxidative stress. The polyol pathway, along with the hexosamine biosynthetic pathway and PKC, becomes activated under hyperglycemia, creating redox imbalance, endothelial dysfunction, and impaired wound healing observed in diabetic foot ulcers (DFUs) [8,32]. Elevated ROS levels act as second messengers to trigger apoptosis in tumor necrosis factor-alpha (TNF-α)-induced processes via NF-κB activation and MAPK inhibition [33].

The mechanisms create a more intense inflammatory environment that leads to ongoing tissue damage. Delayed healing and extended inflammatory responses are common in DFUs. The sustained increase in TNF-α, Interleukin-1 beta (IL-1β), and IL-6 interrupts the proliferative and remodeling stages of healing. Neutrophils are essential during the inflammatory phase, along with monocytes and macrophages. Under healthy conditions, monocytes that reach wound sites differentiate into macrophages, which often shift from pro-inflammatory M1 to pro-resolving M2 types [16]. The M1-to-M2 transition pathway remains hindered by prolonged cytokine exposure and high blood glucose levels. M1 macrophages foster inflammation and tissue breakdown. M2 subtypes (M2a, M2c, and M2d) assist in tissue remodeling, new blood vessel formation, and resolving inflammation [33,34]. M2a and M2c macrophages express CD206 and CD163, respectively, helping with matrix buildup and anti-inflammatory functions. M2d macrophages, activated by toll-like receptor (TLR) ligands, adenosine A2 receptor agonists, and IL-6, produce vascular endothelial growth factor (VEGF) and IL-10, encouraging new blood vessel growth [34]. In diabetes, the process of M2 polarization is blocked, causing persistent inflammation and impaired tissue repair.

The development of DFU is significantly affected by keratinocyte dysfunction. Keratinocytes, the primary cells in the epidermis, are highly vulnerable to AGE-mediated toxicity. Excessive accumulation of AGEs hampers keratinocyte proliferation, survival, and migration by increasing NF-κB activation, raising TNF-α levels, and decreasing ROS-scavenging activity [17,35]. This slows re-epithelialization and causes prolonged disruption of the epidermal barrier. Additionally, the same pro-oxidative and pro-inflammatory environment negatively impacts fibroblasts and endothelial cells, leading to poor granulation tissue formation and impaired angiogenesis [8]. Elevated production of matrix metalloproteinases (MMPs) in chronic wounds degrades extracellular matrix components, hindering tissue regeneration [19]. The functions of M2 macrophages, which produce MMPs and VEGF for tissue remodeling, are impaired because diabetes disrupts the necessary M1-to-M2 transition [33]. Persistent inflammation and ongoing tissue damage further delay the healing process.

**Figure 2:**
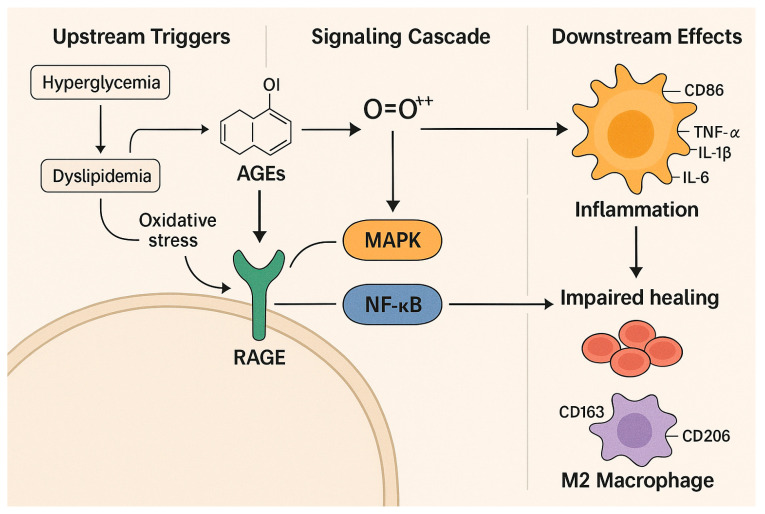
Molecular pathways connecting chronic hyperglycemia to DFU development. Chronic hyperglycemia and dyslipidemia promote AGE formation and oxidative stress, which activate the RAGE-MAPK/NF-κB pathways. These molecular-driven inflammations and disruptions impair immune function, angiogenesis, and M2 macrophage-mediated tissue repair, contributing to DFU formation. These pathways offer potential targets for redox-modulating and anti-inflammatory treatments.

Therapies that focus on restoring redox balance while addressing chronic inflammation show promise for improving DFU outcomes, based on current research. The combination of Nrf2 pathway modulation with AGE–RAGE, protein kinase C, and polyol and hexosamine pathway interventions indicates potential for better DFU results [8,20]. This is depicted in Figure 2.

## 3. The Synergy of Glycemic Control and Antibiotic Therapy in DFU Management

Implementing innovations in glucose monitoring, targeted glycemic goals, bacterial profiles, antibiotic regimens, and antihyperglycemic strategies can lower DFU severity and accelerate healing [4,36,37].

### 3.1. Innovations in Glycemic Monitoring and Control

CGM, introduced in 1999, has transformed glycemic management by providing real-time and predictive data, enabling trend detection and identification of asymptomatic events. Studies show that regular CGM use improves time-in-range and reduces hypoglycemia [36]. Compared to Self-Monitoring of Blood Glucose (SMBG), CGM more reliably captures daily glycemic fluctuations, surpassing the limitations of HbA1c, which cannot reflect short-term variability [38]. Time-in-range refers to the percentage of time blood glucose stays between 3.9 and 10 mmol/L, alongside HbA1c, to predict complications such as DFU severity, diabetic retinopathy, neuropathy, and nephropathy. Yin et al. reported that uncontrolled HbA1c levels and poor perioperative time-in-range are linked to a higher risk of major lower-extremity amputation in DFU patients [38].

A randomized clinical trial showed that CGM significantly lowered HbA1c (from 9.1% to 8.0%) in insulin-treated patients, surpassing traditional blood glucose meters (BGM) [39]. Non-invasive monitoring technologies, such as near-infrared spectroscopy and thermal-based sensors, offer continuous feedback through skin-based measurements [40,41]. These systems enable timely treatment adjustments crucial for DFU management. Emerging closed-loop systems, like Neural CGMM, automate insulin delivery with predictive algorithms but require calibration. Integration with mobile apps and Global System of Mobile Communication (GSM)-enabled glucometers (with 99.9% reliability and 95.1% accuracy) improves data sharing. At the same time, short message service (SMS) alerts enhance patient–provider communication, reducing infection risks [41]. Novel fiber scaffolds embedded with immunomodulatory and antibacterial agents provide targeted DFU treatment by decreasing inflammation, fighting infections, and encouraging tissue regeneration [42]. CGM remains a key therapeutic approach for achieving glycemic targets; these technological advancements allow precise control that can boost antibiotic effectiveness and support integrated DFU management (Section 3.6).

### 3.2. Glycemic Control Target

Glycemic control in DFU management is primarily assessed using HbA1c, along with CGM metrics (such as time-in-range and glucose management indicator) and blood glucose monitoring (BGM). The HbA1c test reflects average glucose levels over the past three months and serves as a clinical benchmark for evaluating glycemic control [43]. A meta-analysis showed that hyperglycemia (fasting glucose ≥ 7 mmol/L and HbA1c ≥ 8%) increases the risk of amputation, while HbA1c levels of 7–8% optimize wound healing in T2DM patients without affecting mortality [44]. However, some studies have found no apparent connection between baseline HbA1c and healing [45], possibly due to different follow-up periods or confounding factors [46]. Despite this, poor glycemic control remains a strong predictor of DFU severity, with higher HbA1c levels linked to increased need for debridement and more extended hospital stays in T2DM patients [47]. In individuals with diabetic foot syndrome, strict glycemic control was associated with a 35% reduction in amputation risk [45]. Hyperglycemia worsens infections by causing insulin resistance and impairing immune function [25].

HbA1c targets of 7–8% are generally ideal for promoting wound healing and reducing complications. A target of <7.0% is appropriate for non-pregnant adults in good overall health, while a stricter goal of 6.5% may be considered for patients without a risk of hypoglycemia [48]. Conversely, a more lenient target of <8.0% is often suitable for patients with limited life expectancy, a history of severe hypoglycemia, or advanced macrovascular or microvascular complications, such as long-standing diabetes or end-stage kidney disease [48].

For patients with stable conditions, testing HbA1c every six months is adequate, while those not meeting treatment goals need testing every three months [43]. However, careful interpretation is necessary when conditions that affect red blood cell turnover, such as anemia, recent blood transfusions, or drugs that promote erythropoiesis [49], are present. Healthcare practitioners should periodically review HbA1c targets, especially in older adults, to customize management strategies effectively [43]. A variety of antihyperglycemic drugs, including insulin, DPP-4 inhibitors, GLP-1 receptor agonists, metformin, and sulfonylureas, can be used to help patients achieve personalized glycemic goals [45]. Although overly strict glycemic control (HbA1c < 7%) has not been shown to improve wound healing and may increase the risk of amputation if hyperglycemia persists (HbA1c ≥ 8%), this indicates that well balanced, patient-specific targets in the 7–8% range are generally safer and more effective [46].

In DFU care, intensive glycemic control is challenging due to limited conclusive data, severe diabetic complications, complicating factors such as infections, and individual patient considerations. Consequently, HbA1c targets of 7–8% are often considered appropriate, as intensive control lacks evidence for healing benefits and is complicated by infections [46]. Rapid and consistent glycemic control is essential for better outcomes [50].

Regular HbA1c testing every three months is recommended for patients with uncontrolled diabetes, while those at target may require less frequent monitoring [47]. Evidence also supports that frequent clinic visits, especially when integrated with wound care services, significantly improve glycemic outcomes. This multidisciplinary approach allows for better coordination between endocrinologists, primary care providers, and wound care specialists, leading to more comprehensive management of DFUs and the underlying metabolic issues that cause them [47].

Apart from preventing neuropathy and vascular problems, maintaining stable glycemic levels improves antibiotic effectiveness, supporting infection management [10]. It demonstrates a synergistic therapeutic relationship: glycemic stability enhances immune function and reduces pathogen virulence, while effective infection control lowers inflammation and helps keep glycemic levels balanced.

### 3.3. Bacteria and Antibiotic Resistance in DFUs

DFUs are typically polymicrobial, with Staphylococcus aureus as the most common isolate, followed by Pseudomonas aeruginosa, which exhibits high multidrug resistance [51]. Other common Gram-positive bacteria include Enterococcus faecalis, Streptococcus pyogenes, and Staphylococcus epidermidis, while Gram-negative bacteria such as Proteus mirabilis, Klebsiella pneumoniae, and Escherichia coli are also frequently found [4,52]. These pathogens often show resistance to antibiotics, including ampicillin, trimethoprim-sulfamethoxazole, levofloxacin, ciprofloxacin, and cefuroxime [4].

The increase in antibiotic self-medication and empiric prescriptions without culture confirmation contributes to inappropriate initial treatment, raising the risk of reinfection, amputation, and death [4]. Poor glycemic control (high HbA1c and blood glucose) and comorbidities like neuropathy and peripheral vascular disease also increase the risk of antibiotic-resistant infections [4]. Studies indicate that uncontrolled HbA1c relates to more severe infections, more extended hospital stays, and a higher need for debridement procedures [47]. In this context, antibiotic failure results from both pharmacological and metabolic challenges. This highlights the importance of integrated strategies that combine strict glycemic control with culture-guided antibiotic therapy to improve outcomes in DFU management [4,51].

Molecular assays like multiplex PCR are increasingly crucial in guiding culture-based antibiotic choices by detecting resistance genes in DFU pathogens. These tests identify extended-spectrum β-lactamase (ESBL) genes, including blaTEM, blaSHV, blaCTX-M, and blaOXA-1, as well as carbapenemase genes such as blaKPC, blaGES, blaOXA-48, blaGIM, blaNDM, blaVIM, and blaIMP [53]. In Escherichia coli isolates, blaTEM is the most commonly detected gene. In Acinetobacter baumannii, resistance often results from ESBLs and metallo-beta-lactamases (MBLs), especially blaIMP, blaVIM, and blaNDM-1 [54]. These gene products lead to high resistance levels against cephalosporins, carbapenems, fluoroquinolones, tetracyclines, and trimethoprim/sulfamethoxazole, strengthening the multidrug-resistant nature of Acinetobacter baumannii [54].

Importantly, resistance can persist even without known resistance genes, through mechanisms such as the loss of porin channels and efflux pump overexpression. In methicillin-resistant Staphylococcus aureus (MRSA), resistance is primarily mediated by the mecA gene. Biofilm formation, present in up to 60% of carbapenemase-positive isolates, further worsens treatment failures by protecting bacteria from antibiotics [55]. These findings highlight the importance of combining molecular diagnostics with phenotypic culture methods to guide targeted antibiotic therapy in DFU and DFI cases.

### 3.4. Association Between Antibiotics and the Severity of DFU in Patients with T2DM

To date, the literature has not provided conclusive evidence supporting the role of antibiotics in speeding up wound healing. However, during the active phase of DFI, antibiotics are necessary to promote healing [56] by targeting pathogens such as Staphylococcus aureus and Pseudomonas aeruginosa. Antibiotics like Linezolid are known to support outpatient management by reducing DFU severity, thereby lowering hospitalization costs. Nevertheless, Linezolid may affect insulin sensitivity and increase the risk of hypoglycemia in patients [56]. A randomized controlled study demonstrated that antibiotic-loaded bone cement is effective for neuropathic DFU and osteomyelitis, reducing DFU severity, the number of operations, wound dressing frequency, and the length and cost of hospitalization [57]. A new series of antibiotics, cefiderocol and dalbavancin, has shown effectiveness in treating severe DFIs caused by multidrug-resistant microorganisms [58]. Although these antibiotics present promising therapeutic options, further research is needed to establish clear guidelines.

### 3.5. Antibiotic Regimen in the Management of DFU

Systemic antibiotics should be used only for DFUs showing clear signs of systemic or local infection, targeting the pathogen identified in Section 3.3. Broad-spectrum antibiotics are advised for moderate-to-severe infections until culture and sensitivity results guide targeted therapy, with treatment for mild soft tissue infections typically not exceeding 14 days [37]. Antibiotics that target Gram-positive bacteria (such as bacitracin and mupirocin) and Gram-negative bacteria (such as neomycin and silver sulfadiazine) are among the options available, both for treating infected wounds and for preventing infection in uninfected wounds [37].

The 2018 Malaysian Clinical Practice Guideline outlines regimens: for mild infections (local erythema less than 2 cm and no systemic symptoms), first-line options include Cephalexin 500 mg orally (PO) every 6 h or Amoxicillin/Clavulanate 625 mg PO every 8 h, with alternatives such as Trimethoprim/Sulfamethoxazole 5–10 mg/kg PO every 12 h or Clindamycin 300–450 mg PO every 8 h, for 1–2 weeks.

Moderate infections (deep tissue involvement, erythema over 2 cm, no signs of systemic inflammatory response syndrome) are treated with Ampicillin/Sulbactam 1.5–3 g administered intravenously (IV) every 6–8 h or Ceftriaxone 1–2 g IV once daily, with Metronidazole 500 mg IV every 8 h as an alternative. Ciprofloxacin 400 mg IV every 8–12 h and Clindamycin 600 mg IV every 8 h are options instead of Piperacillin/Tazobactam. It is recommended to wait 2–4 weeks, or longer if osteomyelitis is present [37]. Severe infections (with systemic inflammatory response syndrome) require Cefepime 1–2 g IV every 8 h or Piperacillin/Tazobactam 4.5 g IV every 6–8 h, with added Vancomycin 1 g IV every 12 h if MRSA is suspected, for 4–6 weeks [37]. For confirmed or suspected MRSA infections, Linezolid 600 mg IV or orally (PO) every 12 h or Vancomycin 15–20 mg/kg IV every 8–12 h is recommended [37].

### 3.6. Role and Molecular Mechanism of Glycemic Control and Antibiotic Therapy in DFU Management

#### 3.6.1. Therapeutic Targeting of DFU Pathophysiology

**Figure 3:**
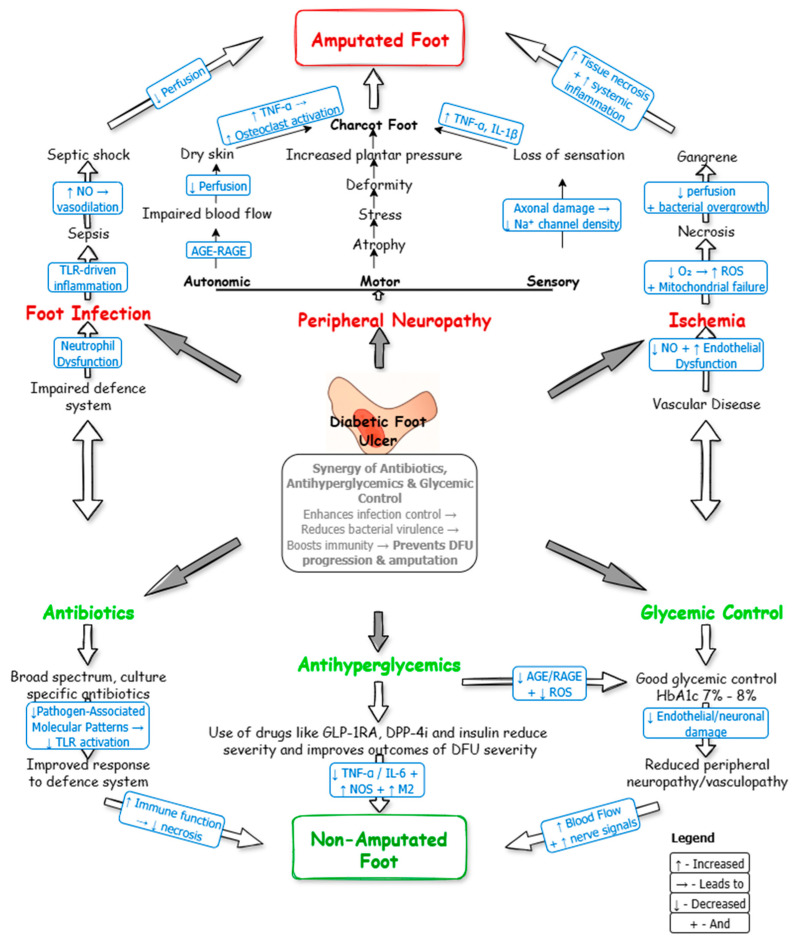
Synergy of antibiotics, antihyperglycemic agents, and glycemic control in managing the severity of DFU. Uncontrolled diabetes results in DFU complications such as peripheral neuropathy, foot infection, and ischemia, which can lead to amputation by activating molecular pathways that cause inflammation and other issues. Conversely, the proper use of antibiotics, antihyperglycemic agents, and glycemic control can prevent this progression by reducing DFU severity and improving outcomes through a series of molecular processes.

DFUs develop through various pathophysiological mechanisms, including infection, peripheral neuropathy, and ischemia, which often lead to serious complications such as amputation (Figure 3). Infections, caused by impaired immune responses, may progress to sepsis or septic shock [59]. While peripheral neuropathy, characterized by motor, autonomic, and sensory dysfunction, contributes to complications like Charcot’s foot [60], ischemia can result in necrotizing fasciitis or gangrene [61]. Effective glycemic control is essential in preventing this pathological progression. Antihyperglycemic agents such as DPP-4 inhibitors (e.g., teneligliptin) and GLP-1 receptor agonists (e.g., liraglutide, semaglutide) have been shown to lessen the severity of neuropathy by improving nerve conduction and reducing oxidative stress [62]. Additionally, these agents promote DFU healing by stabilizing blood glucose levels [63].

#### 3.6.2. Synergy of Glycemic Control and Antibiotics

Antibiotic effectiveness improves with stable glycemic control, which lowers DFU-related infections and enhances immune function. Metformin, beyond its glucose-lowering effects, shows anti-inflammatory, antibacterial, and anti-virulence properties, making it especially useful in DFU management [64]. Reduced bacterial growth and improved immune response are essential for effective DFU treatment. Additionally, the combination of metformin and vildagliptin demonstrates synergistic anti-virulence activity against Pseudomonas aeruginosa, specifically targeting quorum sensing, a key mechanism in bacterial resistance and disease development [65]. This provides a promising approach for treating multidrug-resistant bacterial infections without promoting antimicrobial resistance.

#### 3.6.3. Molecular Mechanism of Combined Therapy

Glycemic control and antibiotic therapy work together in managing DFU, as both influence key molecular pathways involved in inflammation, immune response, and tissue healing. Early evidence suggests a link between controlled HbA1c levels and improved wound healing [66], with one study noting increased macrophage phagocytic activity in diabetic patients after short-term intensive glucose management [67]. Under hyperglycemic conditions, neutrophil dysfunction can occur, characterized by excessive production of ROS that impair immune regulation and contribute to tissue damage and vascular inflammation [68]. Simultaneously, macrophage phagocytic activity may decrease, further weakening antibacterial defenses [69]. These immune changes increase patients’ risk of infections. Additionally, excess hydrogen peroxide, oxidative stress, and related products caused by hyperglycemia promote AGE formation [70]. As a result, maintaining HbA1c at 7–8% would reduce AGE buildup and RAGE activation, helping to prevent NF-κB-driven inflammation [29]. This, in turn, improves bacterial clearance during antibiotic therapy as endothelial function stabilizes [4].

Maintaining stable glucose levels can reduce pro-inflammatory cytokines (e.g., TNF-α, IL-6), potentially fostering a more favorable immune environment that enhances antibiotic effectiveness [4,64]. In addition to glucose-lowering effects, antihyperglycemic drugs like metformin, DPP-4 inhibitors, and GLP-1 receptor agonists also have molecular impacts. Metformin has been shown to activate AMP-activated protein kinase (AMPK) and inhibit the mechanistic target of rapamycin (mTOR) pathway, helping to decrease inflammation and promote autophagy [64]. These actions may support the body’s ability to eliminate bacterial biofilms, though direct evidence in biofilm-related infections remains limited. DPP-4 inhibitors and GLP-1 receptor agonists have been reported to increase nerve growth factor (NGF) and vascular endothelial growth factor (VEGF), which could assist in nerve regeneration and angiogenesis, respectively [62,63]. These effects may enhance blood flow and improve immune cell access to wounds, thereby increasing antibiotic efficacy [64,65].

Besides systemic glycemic control, topical insulin has been shown to accelerate wound healing through various molecular pathways. It promotes the release of VEGF from dermal fibroblasts, enhancing angiogenesis, granulation tissue formation, epithelialization, and wound size reduction [71]. Insulin also activates intracellular pathways, resulting in increased β-catenin expression, which regulates cell proliferation and differentiation for tissue regeneration. Additionally, it suppresses pro-inflammatory cytokines such as TNF-α, improving the inflammatory microenvironment [71].

**Figure 4:**
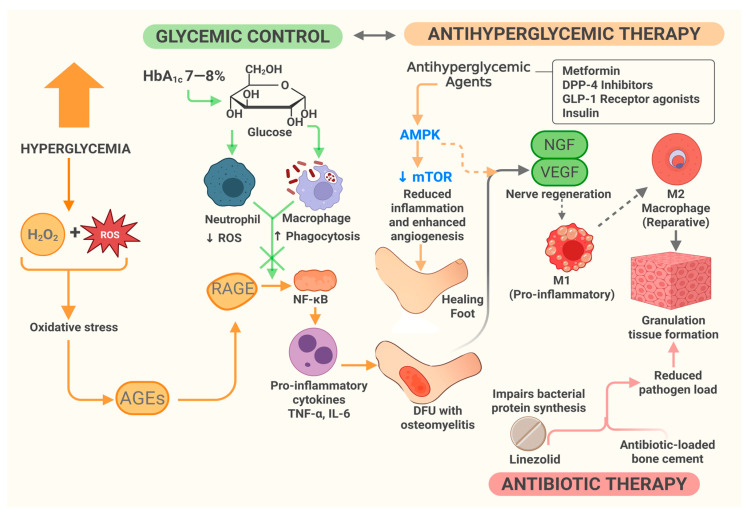
Synergistic molecular mechanisms of glycemic control, antihyperglycemic agents, and antibiotic therapy in diabetic foot ulcer. Hyperglycemia induces increased ROS production, impairing immune regulation and promoting inflammation. Antihyperglycemic agents modulate pathways such as AMPK, mTOR, VEGF, and NGF to improve angiogenesis, nerve regeneration, and tissue repair. Glycemic control decreases AGE–RAGE–NF-κB activation, while antibiotics lower bacterial load and aid in resolving inflammation. Together, these therapies restore immune function and support DFU healing.

Moreover, insulin activates insulin receptors and the PI3K-AKT signaling pathway, enhancing keratinocyte migration and proliferation. In later stages of wound healing, it promotes extracellular matrix remodeling by stimulating fibroblast activity. Additionally, insulin attracts macrophages to the wound site, facilitating efficient debris removal and tissue recovery [72]. Similar to antihyperglycemic agents, antibiotics can exert molecular effects. For example, linezolid, recommended in DFI guidelines for targeting Gram-positive pathogens, works by inhibiting bacterial protein synthesis, thereby reducing pathogen load [73]. To resolve chronic inflammation in diabetic wounds, glycemic control is essential, as it shifts the immune response from pro-inflammatory M1 to reparative M2 macrophages, supporting tissue repair [30]. On one hand, antibiotics reduce bacterial burden; on the other, they help lessen inflammatory signals, further aiding wound healing. Additionally, antibiotic-loaded bone cement delivers high local concentrations of antibiotics, effectively controlling infection and accelerating granulation tissue formation in diabetic foot wounds with osteomyelitis [57]. This is illustrated in Figure 4.

#### 3.6.4. Clinical Staging and Therapeutic Applications

**Figure 5:**
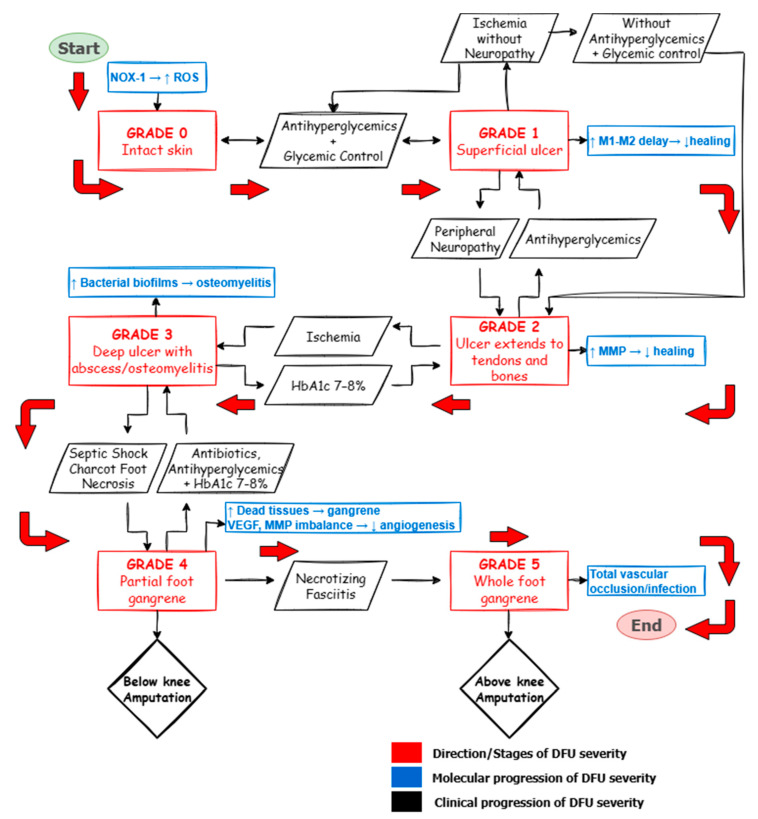
Flowchart showing the severity of DFU and its link with antibiotic treatment, antihyperglycemic drugs, and blood glucose control. DFUs advance from healthy skin (Grade 0) to complete foot gangrene (Grade 5), driven by molecular pathways such as oxidative stress, infection, poor blood vessel growth, and vascular damage. Each stage of DFU severity is connected to specific molecular causes. The reverse arrows also show pathways to recovery using medications like GLP-1 receptor agonists, DPP-4 inhibitors, insulin, antibiotics, and keeping HbA1c within an optimal range (7–8%).

The flowchart in Figure 5 illustrates this synergy—the connection between DFU severity, glycemic control, antihyperglycemic drugs, and antibiotic therapy—using the Meggitt–Wagner classification system. At grade 0 (intact skin), progression to the superficial ulcer (Grade 1) or deeper involvement of bones and tendons (Grade 2), caused by trauma or neuropathy, can often be prevented through early treatment with antihyperglycemic medications (e.g., DPP4 inhibitors, GLP-1 receptor agonists) [74]. A recent population-based study found that DPP-4 inhibitors were associated with a lower occurrence of DFU compared to sulfonylureas (hazard ratio 0.88). Conversely, the use of GLP-1 receptor agonists was linked to a significantly lower risk of DFU compared to insulin, both in short-term (hazard ratio 0.44) and long-term use (hazard ratio 0.74) [75]. These drugs also relate to reduced hospitalization and lower limb amputation rates. For more advanced stages—Grade 3 (deep ulcers with abscess and osteomyelitis) and Grade 4 (partial foot gangrene)—the combination of antibiotics and antihyperglycemic agents significantly lowers the risk of amputation (Grade 5, whole foot gangrene) [76]. Besides controlling blood glucose, incretin-based therapies (GLP-1 receptor agonists and DPP-4 inhibitors) have been shown to improve outcomes in diabetic peripheral neuropathy, a major contributor to DFU progression. Experimental and clinical studies suggest they increase intraepidermal nerve fiber density, Schwann cell function, and nerve conduction velocity, as well as support motor and sensory recovery [77]. These effects make them especially relevant in the early stages of DFU, where neurovascular support is vital for healing. Although the role of Sodium-Glucose Cotransporter 2 inhibitors like Canagliflozin in reducing lower-extremity amputations remains uncertain, some studies suggest a possible link to an increased risk of such amputations [78].

### 3.7. Real-World Challenges and Recommendations for Improving the Outcomes in DFU and DFI

Improving outcomes in DFUs and DFIs requires a comprehensive, team-based approach that addresses both clinical management and the patient’s underlying circumstances. Demographic and socioeconomic factors significantly influence these outcomes. For example, African American diabetic patients living in rural communities in the U.S. face a higher mortality risk and twice the amputation risk compared to non-Hispanic white individuals, mainly due to limited healthcare access [79]. The COVID-19 pandemic disrupted care access for T2DM patients with DFU because of movement restrictions, transportation challenges, and fear of exposure, leading to reduced adherence to treatment; however, no direct increase in amputation or mortality was observed [80].

Comorbidities such as neuropathy, chronic kidney disease, retinopathy, anemia, cardiovascular disease, and peripheral artery disease significantly affect DFU severity and healing outcomes [81,82,83,84,85,86]. For example, chronic kidney disease (eGFR < 60 mL/min) is linked to poor wound healing, higher amputation rates, and increased mortality [83,84]. Immunosuppression, present in 38% of infection (DFI) cases due to conditions like dialysis or cancer chemotherapy, raises the risk of treatment failure (ratio of 1.5), mainly because of wound breakdown rather than failure of anti-infective treatment [87].

Routine use of CGM, as discussed in Section 3.1, should become standard practice to keep HbA1c within 7–8%. This level of control optimizes immune function and improves the effectiveness of antibiotics in lowering infection severity. Targeted antibiotic therapy guided by wound cultures, followed by treatment based on sensitivity results (Section 3.3 and Section 3.5), is vital for preventing resistance, reducing infection severity, and avoiding complications like amputation.

Surgical debridement, appropriate antibiotic therapy, vascular assessment, and offloading with non-removable knee-high devices are crucial for wound healing. When possible, clinicians should include Hyperbaric Oxygen Therapy (HBOT) and Negative Pressure Wound Therapy (NPWT), which further support wound healing, especially when combined with optimal glucose management [88]. Preventive strategies are equally important. These include regular foot inspections [61], patient education on self-care, and early screening for DFU risk factors. High-risk populations and patients with multiple comorbidities need special attention, where personalized, multidisciplinary care models can significantly improve long-term outcomes. Instead of viewing glycemic control and infection management as separate processes, they should be seen as interconnected strategies, with their interdependence increasingly backed by emerging molecular evidence (Section 3.6).

However, several real-world challenges limit the effectiveness of these strategies. Low health literacy reduces regular foot exams and awareness of DFU management, with many patients lacking preventive education before ulcer development [89]. Financial hardship, including costs for insulin, co-payments, and transportation, particularly affects patients in rural areas, where long travel distances and difficulty accessing equipment and supplies can delay medical treatment [89]. Medication adherence also remains suboptimal (53% for α-glucosidase inhibitors, 55% for biguanides, 61% for Sodium-Glucose Cotransporter 2 Enzyme Inhibitors and insulin secretagogues), often due to side effects such as hypoglycemia and gastrointestinal discomfort [90]. Additionally, patients with DFU are frequently prescribed 5–8 concurrent medications, increasing the risk of drug–drug interactions. For example, thiazide diuretics can increase insulin resistance, while heparin, ACE inhibitors, sulfonamides, and salicylic acid may interact with metformin, complicating glycemic control and worsening DFU severity [91,92].

## 4. Conclusions

This review synthesizes emerging molecular insights into how glycemic control, antihyperglycemic therapy, and antibiotics work together to influence the severity and outcomes of DFUs, providing a pathway from control to cure for T2DM patients. At the molecular level, maintaining HbA1c within 7–8% through CGM helps reduce AGE-RAGE and NF-κB signaling, oxidative stress, and pro-inflammatory cytokines, thereby promoting wound healing and enhancing antibiotic effectiveness. Antihyperglycemic agents like metformin, DPP4 inhibitors, and GLP-1 agonists improve these effects through AMPK activation, mTOR inhibition, and upregulation of NGF and VEGF, aiding tissue repair and angiogenesis. Additionally, glycemic stability boosts the efficacy of culture-guided antibiotics by enhancing macrophage and neutrophil function, decreasing bacterial virulence, and disrupting biofilm formation. Combining glycemic and antimicrobial therapies offers a molecularly integrated approach that shifts DFU treatment from merely controlling the disease toward a functional cure by targeting pathogenic processes. Future long-term studies should quantify the combined impact of these synergistic effects on wound healing and guide the development of treatment guidelines. Integrating molecular diagnostics, precise glycemic tools, and personalized antibiotic regimens holds great potential to revolutionize DFU management and significantly lower the risk of amputation.

## Data Availability

The data presented in this study are available from the corresponding authors upon request but are not publicly available due to privacy and/or ethical restrictions.

## Abbreviations

The following abbreviations are used in this manuscript:

AGE	Advanced Glycation End-product
AMPK	AMP-Activated Protein Kinase
BGM	Blood Glucose Monitor
CGM	Continuous Glucose Monitoring
CKD	Chronic Kidney Disease
COVID–19	Coronavirus Disease 2019
DFI	Diabetic Foot Infections
DFU	Diabetic Foot Ulcer
DNA	Deoxyribonucleic Acid
DPP4i	Dipeptidyl Peptidase 4 enzyme inhibitors
eGFR	Glomerular Filtration Rate
ERK	Extracellular Signal-Regulated Kinase
GLP1	Glucagon-like Peptide 1 receptor agonists
GMI	Glucose Management Indicator
HbA1c	Glycated Hemoglobin
IL-1β	Interleukin-1 Beta
IV	Intravenous
JAK	Janus Kinase
MAPK	Mitogen-Activated Protein Kinase
MMP	Matrix Metalloproteinase
MRSA	Methicillin-Resistant Staphylococcus aureus
mTOR	Mechanistic Target of Rapamycin
NADPH	Nicotinamide Adenine Dinucleotide Phosphate
NF-κB	Nuclear Factor-Kappa B
NGF	Nerve Growth Factor
NOX-1	NADPH Oxidase-1
PAD	Peripheral Arterial Disease
PKC	Protein Kinase C
PO	Per Oral
RAGE	Receptors of Advanced Glycation End-products
ROS	Reactive Oxygen Species
T2DM	Type 2 Diabetes Mellitus
TGF-α	Transforming Growth Factor-Alpha
TLR	Toll-Like Receptor
TNF-α	Tumor Necrosis Factor-Alpha
VEGF	Vascular Endothelial Growth Factor
